# Viscosity Reduction and Drag Reduction Performance Analysis of Bionic Excavator Buckets Based on Discrete Element Method

**DOI:** 10.3390/biomimetics9110686

**Published:** 2024-11-09

**Authors:** Guomin Liu, Xuekai Han, Ziyang Wang, Kun Wang, Zhongsong Zhang, Zenan Duan

**Affiliations:** 1 College of Civil Engineering, Jilin Jianzhu University, Changchun 130118, China15554096095@163.com (K.W.);; 2 College of Biological and Agricultural Engineering, Jilin University, Changchun 130022, China; 3 Key Laboratory of Bionic Engineering, Jilin University, Changchun 130022, China

**Keywords:** bionic excavator bucket, viscosity reduction and drag reduction, discrete element method, EDEM

## Abstract

With the aiming of solving problems with the existing ordinary excavator buckets used in the process of operations (such as heavy digging resistance, ease of adhesion, and others), seven types of bionic buckets and a prototype bucket were designed, based on the contractile-state curve of the earthworm head and the contour curve of the pangolin claw toe. The digging processes of the buckets were simulated using the discrete element method. The results show that, compared with the prototype buckets, all seven types of bionic buckets have significant drag reduction effects at the same digging depth, and the drag reduction effects increase with the decrease of digging speed. Among them, the composite bionic bucket-3 has the highest drag reduction rate, of 14.469% when the digging speed is 2 rad/s. At the same digging speed, different buckets disturb the soil particles to different degrees, and the bionic buckets disturb the soil more significantly compared with the prototype buckets. By conducting contact force field analysis for the buckets, it was shown that the bionic corrugated structure brings the bucket surface into incomplete contact with the soil particles, where the contact is on small areas or even on points, so that the relative velocity between the soil and the shovel body increases under the same driving force, which reduces the excavation resistance. This study provides a theoretical and design basis.

## 1. Introduction

In earthmoving operations, the soil adhesion phenomenon of soil touching parts of the earthmoving machinery commonly exists [1], especially in clay- and wet soil environments. The resulting economic losses and energy consumption problems have been somewhat troublesome to experts and scholars in various countries. Research shows that, for excavation of an earth volume of 100 million m^3^, due to soil adhesion caused by additional diesel fuel consumption of up to 500,000 kg in addition to bucket adhesion, the rated shovel loading capacity can be reduced by 20–30% [2], and thus the production efficiency is reduced by more than 30% [3]. Therefore, it is of great significance to solve the soil adhesion problem, for the purposes of both energy saving and emission reduction.

The enhancement of earth-contacting components predominantly employs biomimetic strategies, incorporating structural and surface adaptations inspired by nature. These include biomimetic structural curves, surfaces [4], electro-permeability, and flexibility. The biomimetic structural curve draws inspiration from several biological models: bear claws [5], the paw contour of the oriental mole [6,7], the streamlined body surface of the sturgeon [8], badger teeth outlines [9] and the forelimb teeth contour of the dung beetle [10]. These biological inspirations are integrated to optimize the contour curves of soil-contacting elements, facilitating directional changes of soil particles and enhancing soil stress distribution during soil interaction, thereby reducing resistance. The biomimetic surface design incorporates various natural structures [11], such as the convex hull found on the dorsal plate of the dung beetle’s prothorax, the ridge scales of the pangolin [12,13], and antlion bionic non-smooth surface morphology [14]. Additionally, it includes the abdominal surface of the dung beetle [15], the corrugated structure of ants [16,17], the earthworm’s body surface [18,19], the tortoise’s convex packet structure [20,21], and the furrowed structure of the shark’s skin [22]. These features aim to increase the contact area with soil particles and optimize the distribution of soil stresses, effectively reducing drag. Ren Luquan et al. [23] developed a bionic electro-osmotic bucket based on the electro-osmotic properties of the earthworm’s surface, which demonstrated substantial viscosity reduction and soil removal capabilities in experimental validations. Similarly, Chen Bingcong and colleagues designed a bionic bucket featuring flexibility characteristics of the earthworm’s surface, achieving a soil removal rate of 59.4%. Furthermore, Sun Shiyuan and associates introduced a bionic steel fabric bib bucket that leverages flexural deformation properties, significantly reducing soil adherence compared to conventional buckets. According to expert research, applying a coating to the surface of earth-contact structures can significantly reduce soil adhesion [24,25].

Prior research on excavator buckets has primarily examined the interaction between bucket components and soil, as well as their operational modes [26]. Contemporary advancements focus predominantly on enlarging traditional components, with limited exploration into novel bucket designs. This study addresses the shortcomings of conventional excavator buckets, such as high digging resistance and material adherence.

The main contents of this study are as follows: (1) By integrating the contraction curve of the earthworm head and the contour curve of the pangolin claw toe, this research optimizes the design of the bucket’s bottom and side plates. (2) A virtual soil trench model was established to simulate the excavation process, enabling the calculation of excavation resistance for various bucket designs and their comparison with standard buckets to determine drag reduction rates. (3) Additionally, the discrete element method was employed to simulate the mechanical behavior of soil particles in interaction with different buckets and their movement. (4) This study also analyzes the drag reduction mechanism facilitated by the bionic ripple structure on the surface, providing a theoretical basis for reducing viscous drag in excavator bucket design.

## 2. Materials and Methods

### 2.1. Materials: Explanation of the Materials and Their Properties

**Figure 1:**
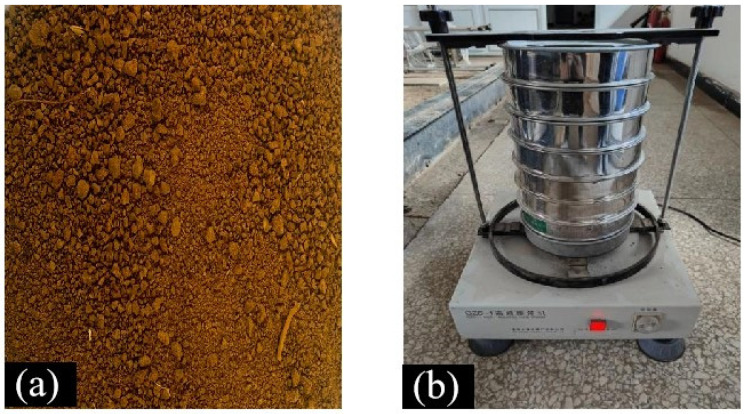
Soil particle size calibration test. (**a**) Test soil, (**b**) high frequency vibrating screen.

Firstly, we measured the properties of the soil, and soil particle parameters were calibrated as shown in Figure 1a. Table 1 shows the distribution of soil particle size. The soil particle size distribution was first determined, using the high-frequency vibrating screen depicted in Figure 1b to calibrate the size distribution. 

**Figure 2:**
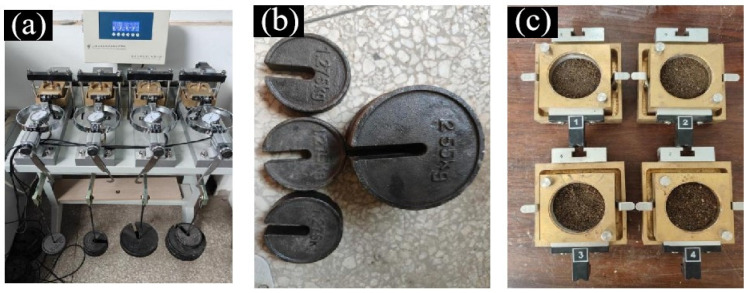
Soil direct shear test. (**a**) Direct shear apparatus, (**b**) loading weight, (**c**) soil preparation.

Additionally, the shear modulus of the soil particles was determined through direct shear tests, as shown in Figure 2. The tests used a ZJ-type strain-controlled direct shear apparatus (as shown in Figure 2a), with a shear rate of 0.800 mm/min and a shear failure time of 3 min. Vertical pressure levels (loading weights depicted in Figure 2b) were set at 50 kPa, 100 kPa, 200 kPa, and 300 kPa. After loading, the sample was left to rest for 2 min to reach a new equilibrium before the commencement of the shear test.

### 2.2. Analysis of Bionic Prototype

**Figure 3:**
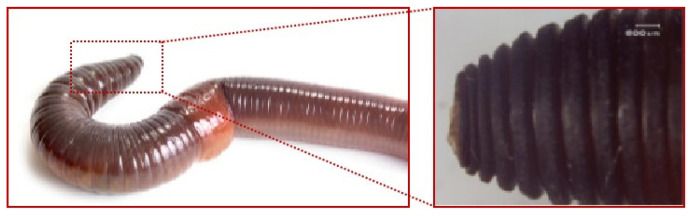
Bionic prototype-structure of the head of an earthworm contracted.

Soil-dwelling animals have developed specialized features through natural selection to minimize the adhesion of earth particles in soil environments. These adaptations allow them to navigate sticky and moist soils effectively without significant soil attachment. Such capabilities are crucial for designing excavator buckets aimed at reducing viscosity and enhancing soil removal. Earthworms, as exemplary soil inhabitants, demonstrate notable efficiency in viscosity reduction and soil desiccation due to a combination of factors including their surface structure, flexibility, lubrication, electro-osmotic properties, and unique movement patterns [27]. Research by Professor Li Jianqiao’s team at Jilin University [28] indicates that the corrugated surface of earthworms significantly contributes to both viscosity and drag reduction. The effectiveness of these features varies by state, with the contractile state offering the greatest reduction, followed by the resting and then the diastolic states; additionally, the head region exhibits a superior drag reduction compared to the body. Consequently, this study focuses on the contractile-state curve of the earthworm’s head [26,29], as illustrated in Figure 3. Image processing was performed using 24-bit RGB greyscale conversion in R2V software 5.5, followed by importation into CAD 2019. Curve fitting was executed in Matlab R2018a, with the resultant equations presented as Equations (1) and (2),

(1) $$\;f\;(x_{1})=0.4sinx$$

(2) $$f\;(x_{2})=0.25sin2x$$

where f (x_1_) is the front part of the contractile-state curve of the earthworm head, and f (x_2_) is the middle and back part of the contractile-state curve of the earthworm head.

**Figure 4:**
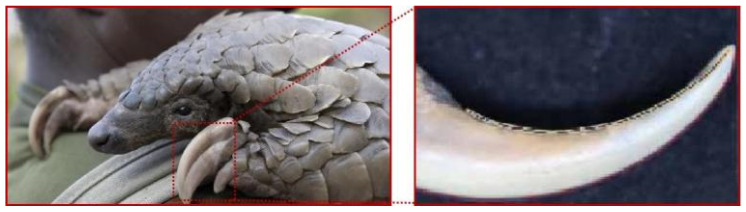
Bionic prototype-structure of the toe of the pangolin claw.

The pangolin, a specialized soil-digging animal, has evolved claw toes adapted for efficient soil excavation over extended evolutionary periods. Figure 4 illustrates the outline of the pangolin and its claw toes. The contours were digitized and processed to obtain fitted curves. The equations representing these fitted curves are detailed in Equations (3) and (4),

(3) $$f\;(x_{3})=-0.139x^{2}+0.91x+0.65$$

(4) $$\;f\;(x_{4})=-0.128x^{2}+1.08x+0.95$$

where f (x_3_) is the outer contour curve of the pangolin claw, and f (x_4_) is the inner contour curve of the pangolin claw.

The characteristics of the different geometries required for testing are detailed in Table 2.

### 2.3. Design of Bionic Bucket

**Figure 5:**
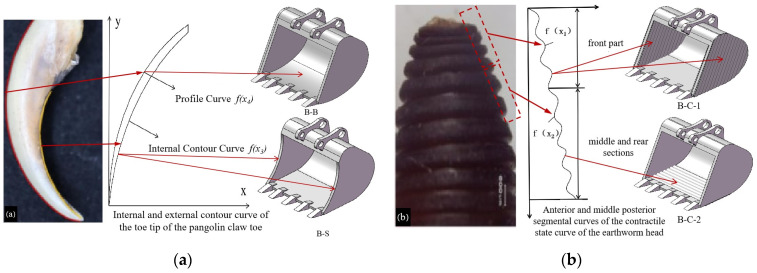
B-S, B-B, B-C-1, and B-C-2 buckets. (**a**) Bionic Bucket Design Based on Pangolin Claw Toe Structure, (**b**) Bionic bucket design based on the corrugated body surface of earthworms.

This paper examines the contact characteristics of different bucket surfaces with the soil interface to ensure valid comparisons. Utilizing the parameters from the aforementioned fitted curves, surface reconstruction was performed using SolidWorks 2021 software. The side plates, bottom plates, and side blades of the bucket were modeled as fitted surfaces, resulting in the design of seven types of bionic buckets alongside a comparative prototype. The outer contour curve *f* (*x*_3_) of the pangolin claw toe was applied to the lateral edge curve of the bucket to create a bionic side-blade bucket (B-S). The toe in-profile curve *f* (*x*_4_) was used for the bucket’s base plate to develop a bionic bottom bucket (B-B). The anterior segment *f* (*x*_1_) of the earthworm’s head in its contractile state was used on the inner and outer walls of the bucket’s side panels to construct a bionic corrugated bucket-1 (B-C-1). Additionally, the mid-back segment *f* (*x*_2_) of the same curve was employed on the upper and lower sides of the bucket base plate to produce a bionic corrugated bucket-2 (B-C-2). These four bucket designs are illustrated in Figure 5.

**Figure 6:**
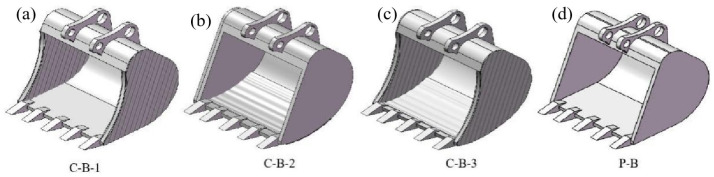
Coupling bionic bucket and prototype bucket. (**a**) C-B-1 bucket, (**b**) C-B-2 bucket, (**c**) C-B-3 bucket, (**d**) P-B bucket.

Upon developing the fundamental bionic bucket designs, this study progresses by integrating distinct bionic features from various designs to enhance functionality. The rounded side-blade features from the bionic side-blade bucket (B-S) are merged with the corrugated features of the bionic corrugated bucket-1 (B-C-1) to create the coupled bionic bucket-1 (C-B-1). Similarly, the circular arc feature of the bionic bottom bucket (B-B) is combined with the corrugated feature of the bionic corrugated bucket-2 (B-C-2), resulting in the coupled bionic bucket-2 (C-B-2). Subsequently, the integrated bionic features from C-B-1 and C-B-2 are amalgamated to form the coupled bionic bucket-3 (C-B-3). Alongside these, a prototype bucket (P-B) is employed to assess the viscosity and drag reduction effects under conditions of single-factor bionic and multi-factor coupled bionic interventions. Figure 6 illustrates both the coupled bionic buckets and the prototype bucket.

## 3. Discrete Element Simulation Modeling

To investigate the excavation resistance, torque, and soil movement characteristics of various buckets, simulations were conducted using EDEM 2021 software on eight distinct bucket designs. The outcomes from these simulations at varying digging speeds were compared and analyzed to evaluate the macroscopic drag reduction effects across different buckets. Additionally, a microscopic analysis was performed on the response of soil particles when in contact with the corrugated surfaces and bionic arc structures of the buckets.

### 3.1. DEM Soil Modeling

Given the discrete nature of soil, EDEM software provides accurate simulation capabilities for bucket excavation processes [30,31], and the discrete element method can effectively characterize the interaction between soil and soil-contacting parts [32,33]. In establishing the discrete element model of soil, it is essential to consider not only the radius and graded sizes of soil particles but also the adhesion between particles under varying moisture content. Due to the adhesion between soil particles caused by water and chemical substances, simplified contact models struggle to accurately simulate the mechanical behavior of the viscous soils commonly encountered in production. Discrete element simulations of soil dynamics using three-dimensional cohesive particle models and wet particle models that account for soil moisture will advance the microscopic analysis of soil dynamics and aid in the innovative design of soil contact components. The selection of the contact model greatly influences the accuracy of simulation results.

**Figure 7:**
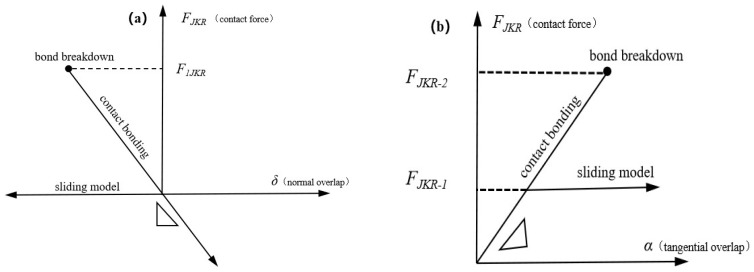
Contact bonding model: (**a**) normal overlap, (**b**) tangential overlap.

The Hert–Mindlin with JKR (Johnson–Kendall–Roberts) contact model, a bonding particle contact model based on the JKR theory where the mutual attraction between particles is represented by surface energy, is employed for this purpose. As depicted in Figure 7, this model effectively approximates the actual contact area force distribution, incorporating the effects of cohesion among wet particles during particle movement. It is adept at characterizing the mutual attraction between particles, making it particularly suitable for materials with a presence of moisture or electrostatic forces, such as soil with certain humidity levels [34,35,36,37]. Consequently, this paper adopts the Hert–Mindlin with JKR soil contact model to analyze the interactions under these conditions, referencing the JKR theory [38].

(5) $$F_{JKR}=\frac{4E^{*}}{3R^{*}}\delta^{3}-4\delta^{3/2}\sqrt{\pi \gamma E^{*}}$$

The equivalent contact radius (R*) and equivalent elastic modulus (E*) are defined as:

(6) $$\frac{1}{E^{*}}=\frac{1-\gamma^{2}}{E_{1}}+\frac{1-\gamma^{2}}{E_{2}}\frac{1}{R^{*}}=\frac{1}{R_{1}}+\frac{1}{R_{2}}$$

where E_1_, v_1_, R_1_ and E_2_, V_2_, R_2_ are the Young’s modulus, Poisson’s ratio, and the radius of the two contact bodies, respectively.

(7) $$\delta =\frac{\alpha^{2}}{R^{*}}-\sqrt{\frac{4\pi \gamma \alpha }{E^{*}}}$$

where F_JKR_—contact force (N); γ—surface energy (J/m^2^); δ—normal overlap (mm); and α—tangential overlap (mm).

The model provides a method for calculating the cohesive force between particles, allowing them to attract each other even without direct contact. The maximum gap between particles with non-zero cohesion is calculated using the following formula:

(8) $$\delta_{c}=\frac{{\alpha_{c}}^{2}}{R^{*}}-\sqrt{\frac{4\pi \gamma \alpha_{c}}{E^{*}}}$$

(9) $$\alpha_{c}=\left[\frac{9\pi \gamma R{*}^{2}}{2E*}\left(\frac{3}{4}-\frac{1}{\sqrt{2}}\right)\right]$$

where *δ_c_* is the maximum normal gap (m) when there is non-zero cohesion between particles, and *α_c_* is the maximum tangential gap (m) when there is non-zero cohesion between particles. When *δ < δ_c_*, the model returns 0. The following diagrams illustrate normal and tangential contacts.

When particles are not in actual contact and the gap is less than δc, cohesion reaches its maximum. The maximum cohesion *F_pullout_* for particles not in actual contact is calculated using the following formula:

(10) $$F_{\mathrm{pullout}}=-\frac{2}{3}\pi \gamma R^{*}$$

### 3.2. DEM Calibration

**Figure 8:**
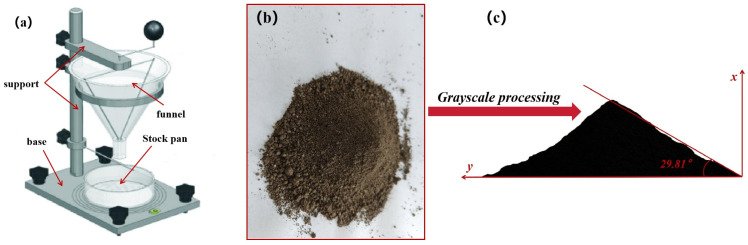
Test deposit angle. (**a**) Funnels, (**b**) test soil, (**c**) test stacking angle measurement.

The angle of repose from the experiment was calibrated against the simulated angle of repose to determine the soil–soil collision recovery coefficient, rolling recovery coefficient, static friction coefficient, and the soil–steel collision recovery coefficient, rolling recovery coefficient, and static friction coefficient. To ensure the accuracy of the simulation, the experimental angle of repose was calibrated against the simulated angle of repose as shown in Figure 8, with the results of the experimental angle of repose presented in Table 3.

**Figure 9:**
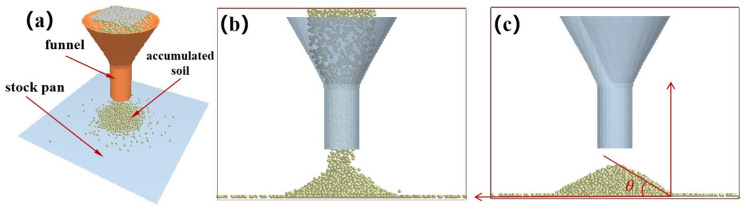
Measurement of simulated pile angle. (**a**) Simulated stacking angle measurement, (**b**) landing process of soil particles, (**c**) measurement process.

When the simulated angle of repose matches the experimental angle of repose, the soil–soil collision recovery coefficient, rolling recovery coefficient, static friction coefficient, and the soil–steel collision recovery coefficient, rolling recovery coefficient, and static friction coefficient are determined. During simulation, initial parameters for the soil model were selected based on literature data and recommended values from the discrete element model database within EDEM software, with the simulated angle of repose depicted in Figure 9 and shown preliminarily in Table 4.

Using Design-Expert 11 software, a Plackett–Burman experimental design was conducted and analyzed with the angle of repose as the response variable. Different parameter combinations yielded various angles of repose in EDEM, across twelve groups of angle of repose simulation experiments. The experimental design and results are shown in Table 5.

From the simulation results, it is evident that the values for the experimental angle of repose fall between Group 3 and Group 10, and analysis shows that Groups 3 and 10 have the same values for factors A, C, and G, but differ in values for B, D, E, and F. Therefore, factors B, D, E, and F were set as variables, and A, C, and G as constants, with the angle of repose as the response value for the Box–Behnken experiment. The factor levels for the Box–Behnken experiment are shown in Table 6.

Table 7 presents the design and results of the Box–Behnken experiment, from which it can be inferred that the angle of repose from the 15th group simulation, 29.68°, is closest to the experimental result of 29.81°. Hence the 16th group data was selected for the soil particle parameters. The specific soil particle parameters used in this simulation are detailed in Table 8.

### 3.3. Soil Trench Modeling

The bucket model was developed using SolidWorks and saved as an IGS file for import into the EDEM simulation software. The mesh of the bucket was refined to 1.5 times the minimum particle radius to ensure accurate interaction with the soil particles. The size of the simulated particles in the discrete element determines the computational efficiency of the simulation, and the reduction of simulated particles increases the number of particles, which greatly increases the computational time and storage space [39]. Through the measurement of soil particle size, it was found that the particle size >2 mm accounted for 46.71%, due to the different and extremely complex shapes of the particles in the soil, which can be mainly divided into massive, columnar and nuclear structures. So, in order to reasonably and efficiently improve the simulation efficiency and balance the simulation accuracy and simulation time, the single, double, and triple spheres particles of 3 mm were selected, and the total number of soil particles was 80,000. The ratio of the three particles was synchronized in the ratio of 5:3:2.

**Figure 10:**
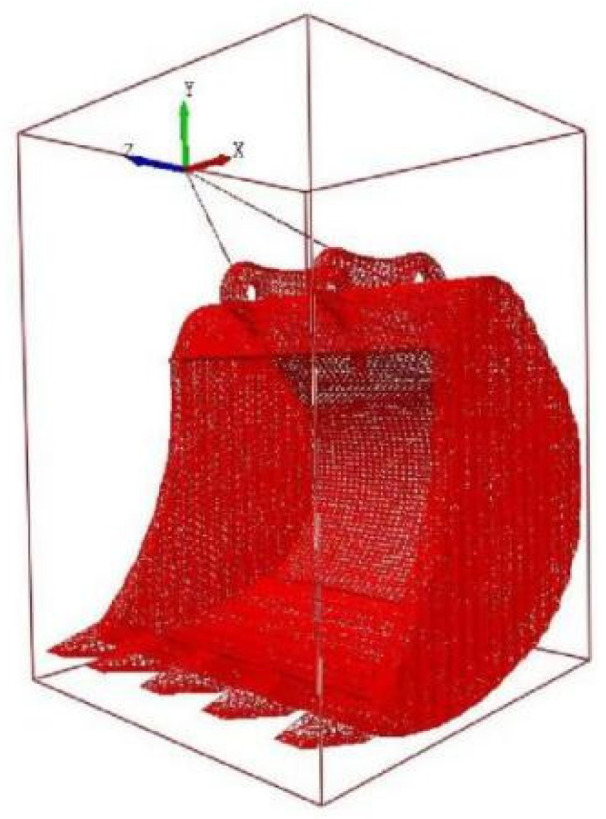
Bucket model.

**Figure 11:**
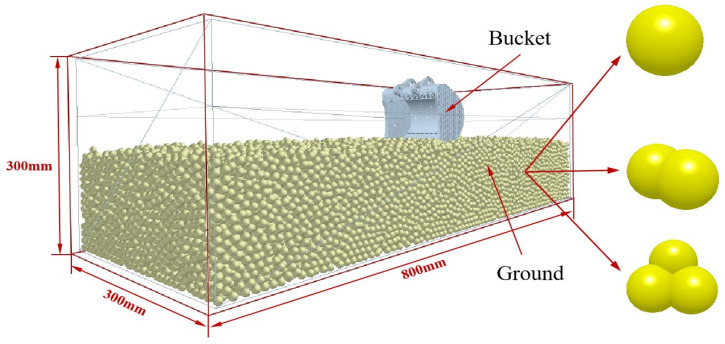
Soil tank model.

**Figure 12:**
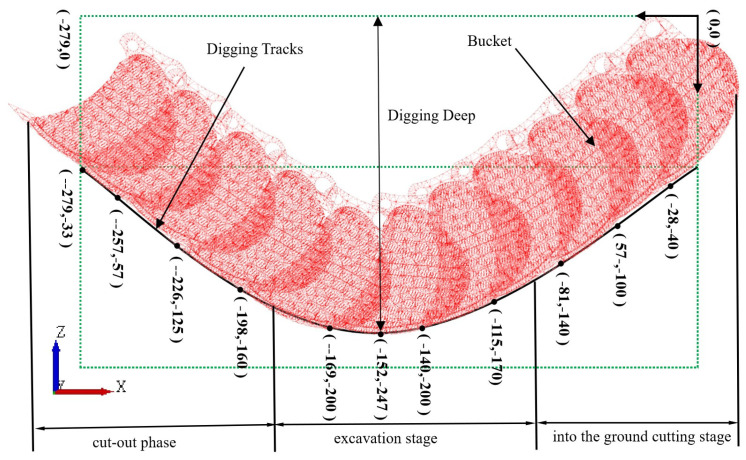
Sketch of bucket excavation trajectory.

Figure 10 displays the bucket model. Utilizing EDEM 2021 software, a soil tank with dimensions of 800 mm by 300 mm by 300 mm was constructed. During pre-processing, the Bulk Material option was used to save and set the soil parameters within the EDEM soil tank model. The soil–bucket interaction simulation was established using the Add Block Factory feature, setting the simulation time step to 25% and conducting data acquisition at intervals of 0.001 s. The virtual soil was generated through natural accumulation within a period of 0 to 2.0 s, followed by a bucket movement phase from 2.0 to 3.0 s, as shown in Figure 11. The simulation model of the soil trough was then used to perform tests on seven bionic buckets and one prototype bucket to evaluate their drag reduction capabilities. Given the dimensions of the virtual soil slot and the operational requirements of the excavator bucket, the digging speed was set to 2 rad/s, 2.5 rad/s, and 3 rad/s, with a constant digging depth of 247 mm to avoid ‘exploding’ soil particles due to excessive speed, which could otherwise increase simulation time. In the pre-processor module of EDEM, the bucket’s digging parameters were configured to ensure consistent depth. Figure 12 illustrates the schematic of the bucket digging trajectory.

## 4. Simulation Results and Analysis

### 4.1. Effect of Digging Speed on Work Resistance

**Figure 13:**
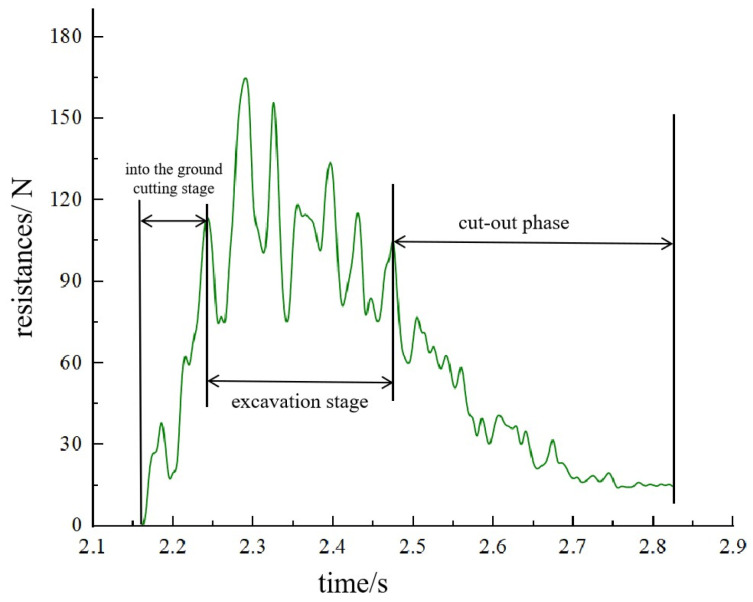
Coupled bionic Bucket-3 (C-B-3) excavation resistance at each stage.

To elucidate the dynamic trend of bucket resistance during different phases of the excavation process, the activity is segmented into three stages: the incoming cutting stage, the excavation stage, and the cutting-out stage. The coupled bionic bucket-3 (C-B-3) was chosen for analysis, as the resistance trends of the buckets are generally consistent. As depicted in Figure 13, under the conditions of a digging speed of 2.0 rad/s and a depth of 247 mm, the discrete element simulation shows that, during the soil-cutting stage, the excavation resistance increases significantly as the contact area between the bucket and the soil expands, reaching a peak at a specific moment. During the excavation stage, the resistance exhibits varying degrees of fluctuation. In the cutting-out stage, the cohesive bond within the soil progressively weakens and ultimately fails, leading to a softening of the soil, reduction in strength, and a consequent gradual decline in excavation resistance.

Table 9 presents the average values of digging resistance and the corresponding reduction rates for the bionic bucket and the prototype bucket at various digging speeds. It indicates that, at speeds of 2.0, 2.5, and 3.0 rad/s, the bionic bucket exhibits lower digging resistance than the prototype. Among the designs, the coupled bionic bucket shows the lowest resistance, particularly the coupled bionic bucket-3 (C-B-3), which has the smallest resistance. Additionally, a trend of decreasing drag reduction rates with increasing digging speeds is observed. Table 10 provides the average digging torque values for both buckets at different speeds, highlighting that the bionic bucket consistently registers lower torque values than the prototype, with the coupled bionic bucket demonstrating significantly reduced torque compared to the prototype 14.

**Figure 14:**
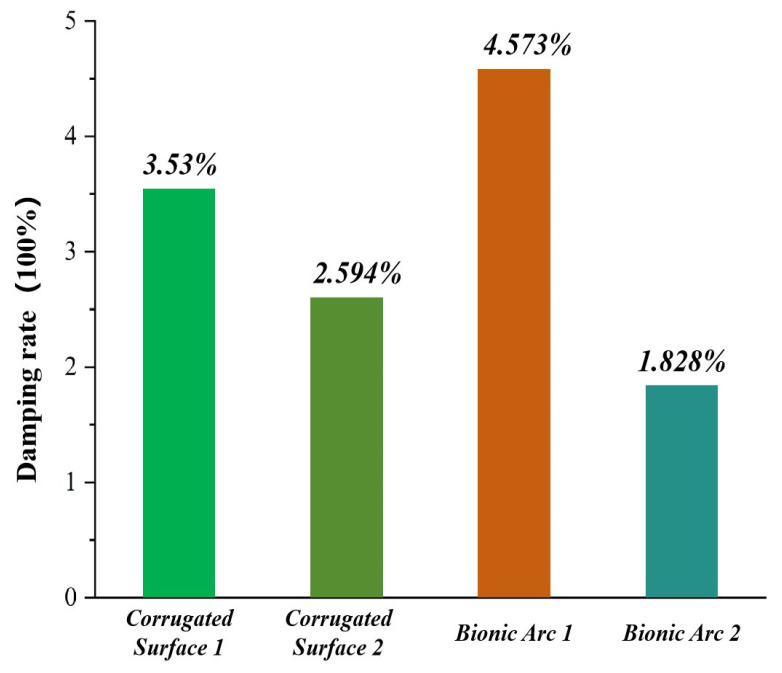
Contribution of corrugated surfaces and circular structural mechanisms to the value of drag reduction.

In this simulation test, a total of two drag reduction mechanisms existed, namely, the bionic corrugated surface and the bionic circular arc structure. Among them, there were two types of bionic corrugated surfaces, Corrugated Surface 1 applied to the B-C-1 bucket and Corrugated Surface 2 applied to the B-C-2 bucket, and there were two types of bionic arc structures, Corrugated Surface 1 applied to the B-B bucket and Corrugated Surface 2 applied to the B-B bucket. Bionic Arc 1 and Bionic Arc 2 were applied to the B-C bucket, and their contributions to drag reduction were 3.53%, 2.594%, 4.573%, and 1.828%, respectively, as shown in Figure 14.

An analysis of simulation data from Table 9 and Table 10 demonstrates that the bionic bucket, designed with a contour curve mimicking the toe tip of a pangolin claw and a curve resembling the contracted state of an earthworm head, effectively reduces drag. This reduction is attributed to the bionic structure’s ability to disperse soil pressure, lessen stress concentration, and enhance soil stress distribution during penetration. Moreover, the bucket’s corrugated and non-smooth surface structure facilitates the formation of an air film at the soil–contact interface. This air film disrupts the water film between the soil and the bucket walls, altering the actual contact area and thereby decreasing resistance, which contributes to drag reduction.

Comparative analysis of combined digging resistance and torque values for various buckets indicates that bionic buckets achieve substantial drag reduction. Specifically, coupled bionic buckets exhibit superior drag reduction compared to single-factor bionic buckets. Notably, the coupled bionic bucket-3 (C-B-3) demonstrates the most effective drag reduction.

### 4.2. Effect of Excavation Speed on Soil Potential Energy

**Figure 15:**
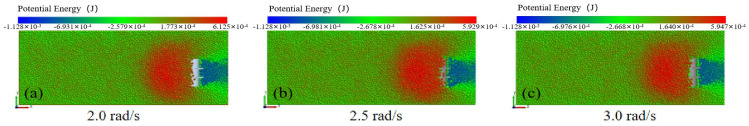
Diagram of potential energy effect of excavation speed on soil particles. (**a**) Soil potential energy at an excavation speed of 2.0 rad/s, (**b**) Soil potential energy at an excavation speed of 2.5 rad/s, (**c**) Soil potential energy at an excavation speed of 3.0 rad/s.

**Figure 16:**
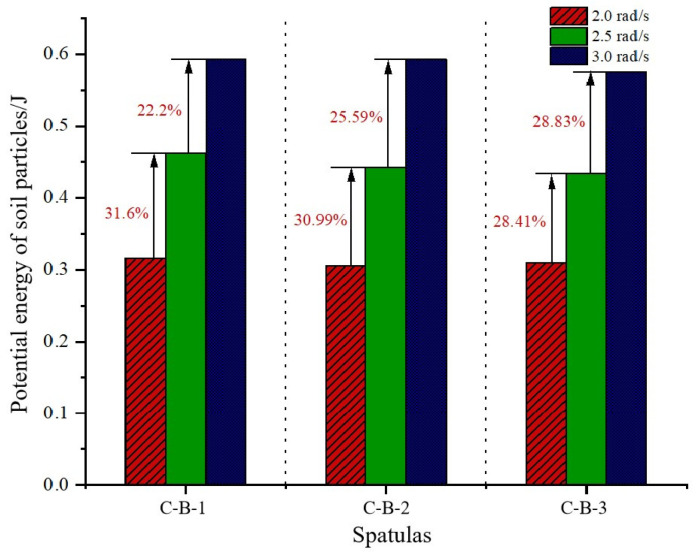
Comparison of kinetic energy of loam particles under different excavation speeds.

When analyzing the drag reduction performance of bucket excavation, the change in potential energy of the soil particles (caused by their displacement relative to the original position during the excavation process) can indicate the effectiveness of the drag reduction. Under varying digging speeds, the bucket exhibits distinct influence patterns on the soil’s potential energy. Analysis reveals that the coupled bionic bucket-3 (C-B-3) demonstrates the most effective drag reduction; hence it has been chosen for detailed examination. Figure 15 compares the impact of different speeds on the potential energy of soil particles with the coupled bionic bucket-3 (C-B-3). The data indicate that, as digging speed increases, the maximum potential energy values of soil particles in contact with the bucket are 6.125 × 10^−4^ J, 5.929 × 10^−4^ J, and 5.947 × 10^−4^ J, respectively, reflecting an increasing trend. This increase is attributed to the enhanced disturbance caused by the bucket to the soil particles as the digging speed escalates. With its superior drag reduction properties, the coupled bionic bucket is used for further analysis. As depicted in Figure 16, at a digging speed of 2.5 rad/s, the potential energy shows an approximate 30% increase compared to at 2.0 rad/s. At 3.0 rad/s, there is a further 25% increase in potential energy relative to at 2.5 rad/s.

### 4.3. Effect of Different Buckets on Soil Disturbance Conditions

**Figure 17:**
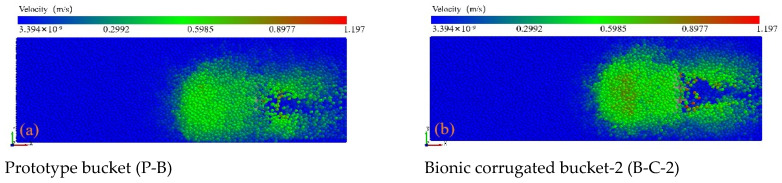

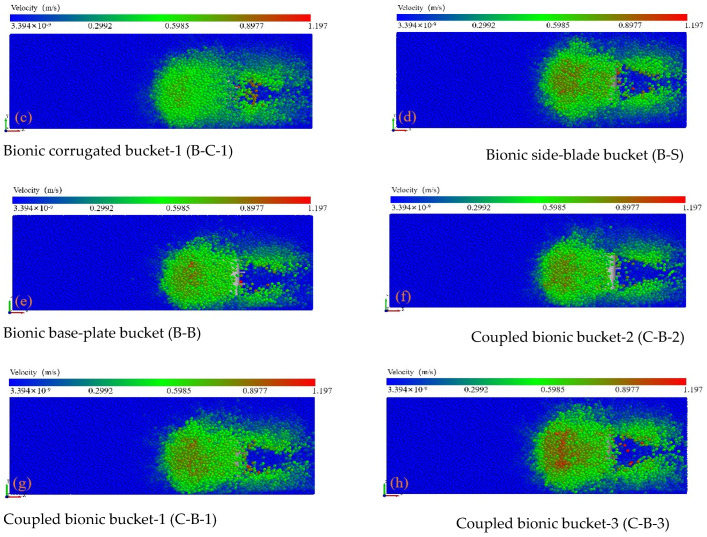
Comparison of soil particle disturbance caused by different buckets.

To observe the intensity and range of soil particle motion more directly, a particle velocity field was introduced to analyze changes in soil particle motion velocity, with particles color-coded based on their velocity. Figure 17 demonstrates that the soil particle motion velocity associated with the bionic bucket is significantly higher than that of the prototype bucket. This increase is attributed to the bionic corrugated surface, which accelerates soil block movement under the same driving force compared to the prototype bucket’s surface. Additionally, the design of the bionic bucket’s bottom plate and side edges effectively disperses soil pressure and alters the direction of soil particle movement at the force’s front end. When compared to the single-factor bionic bucket, the composite bionic bucket, particularly the coupled bionic bucket-3 (C-B-3), exhibits enhanced soil particle perturbation.

### 4.4. Bucket Contact Force Field Analysis

**Figure 18:**
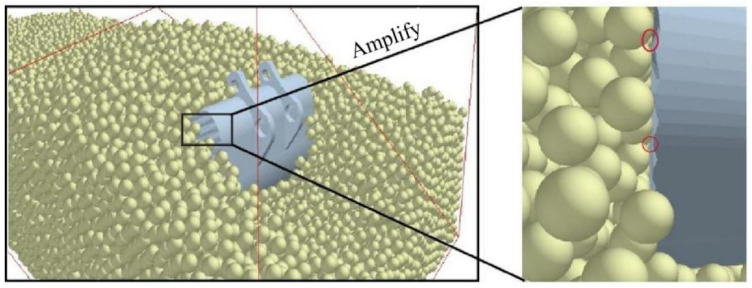
The interface between a corrugated surface and soil.

**Figure 19:**
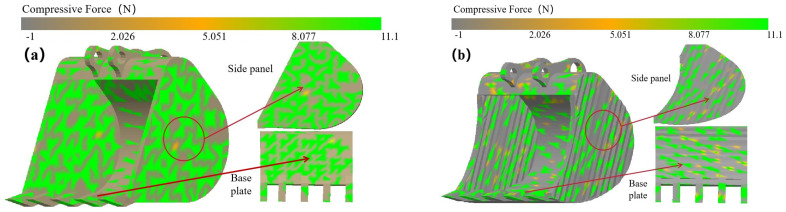
Contact field between bucket and soil particles. (**a**) Prototype bucket contact force field analysis, (**b**) coupled bionic bucket-3 contact force field analysis.

Micro-analysis of the bucket–soil contact surface enhances our understanding of bucket interaction dynamics. The corrugated surfaces on the bucket’s side and bottom plates feature concave and convex ripples, which make incomplete contact with the soil particles, as illustrated in Figure 18. This configuration reduces the actual contact area between the soil and the bucket surface, thereby increasing the mobility of soil particles on the shovel surface—that is, the relative velocity between the soil and the shovel body increases. Consequently, the residence time of soil on the bionic bucket is shortened under the same driving force, effectively reducing excavation resistance. For instance, a comparative analysis of the contact force (compressive force) between the prototype bucket and the coupled bionic bucket-3 (C-B-3) is depicted in Figure 19. It reveals that the coupled bionic bucket-3 (C-B-3) interacts with soil particles in small patches or even experiences point disintegration, whereas the prototype bucket maintains a larger contact area.

### 4.5. Analysis of Corrugated Surface Drag Reduction Mechanism

**Figure 20:**
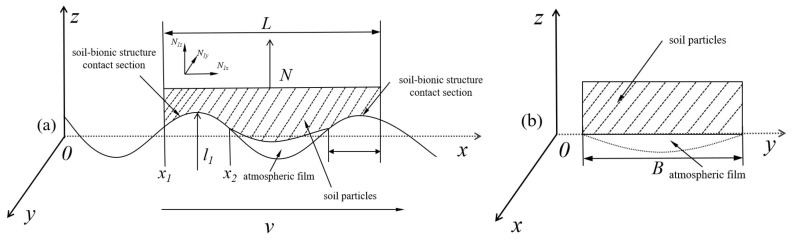
Diagram of corrugated surface in contact with soil. (**a**) Corrugated surface damping mechanism, (**b**) smooth surface contact.

A primary mechanism for the drag reduction on the bionic corrugated surface is the formation of an air film at the contact interface between the soil and the bucket. The corrugated surface features bumps that facilitate the rapid passage of soil particles across the contact area, substantially decreasing the contact area. This reduction in contact area consequently lowers both viscosity and drag. The interaction between the corrugated surface and the soil is illustrated in Figure 20. Here, L represents the contact length between the prototype bucket surface and the soil; N denotes the bucket’s resistance (in Newtons); and *N*1*x*, *N*1*y*, and *N*1*z* are the components of N in the xyz directions. Assuming that the soil contact with the corrugated surfaces of the bucket’s side and bottom plates is equal, the corrugated structures are described by the mathematical functions, f (x_1_) = 0.4sinx, f (x_2_) = 0.25sin2x, both of which are of the form f (x) = Asin (wx). Thus, *l*_1_ is half the length of the contact area of the bionic corrugated bucket with the soil.

In the *xoy* coordinate system, a piece of soil with dimensions *L × B* is established, where *B* is the length in the y direction, and the bucket moves along the x direction. For the bionic earthworm corrugated curve, the bionic structure’s actual contact area with the soil is given by the product of the arc length *l*_1_ and the width *B* of the soil piece. The actual contact area corresponds to the function of the two curve segments described by f (x) = Asin (wx). Assuming the length is *2l*_1_, the bionic contact area between the corrugated bucket and the soil is:

(11) $$\begin{array}{cc} A_{s1} & =2l_{1}B\\ & =2\int_{x_{1}}^{x_{2}}\sqrt{1+f\prime^{2}(x)}B \end{array}$$

The resistance generated by N primarily consists of two components: the adhesion force and the friction force between the soil mass and the bucket surface, collectively referred to as the adhesion friction force. Defining the tangential adhesion force per unit area as *τ_x_*, the adhesion force on the bucket surface for sticky wet soil is given by:

(12) $$F_{ad}=\tau_{x}A_{1s}$$

Due to the large relative motion between the soil mass and the bucket, the friction component of *N* can be expressed as:

(13) $$F_{f}=\mu_{f}N_{1x}$$

where *μ_f_* is the coefficient of friction between the sticky wet soil and the bucket surface.

Then the force *F_bi_* between the surface of the bionic corrugated bucket and the soil during the whole excavation process can be expressed as:

(14) $$\begin{array}{ll} F_{bi} & =2(F_{ad}+F_{f})=2\tau_{x}A_{1s}+2\mu_{f}N_{1x}\\ & =2\tau_{x}l_{1}B+\mu_{f}N\\ & =\tau_{x}lB+\mu_{f}N \end{array}$$

The force *F_pr_* between the surface of the prototype bucket and the soil can be expressed as:

(15) $$F_{pr}=2(F_{ad}+F_{f})=\tau_{x}LB+\mu_{f}N$$

Obviously, l < L. Assuming the bucket moves at a speed *v = v*_0_ while driving the soil block, there is a relative displacement between them. Let the movement speed of the soil unit be v = v_1_, and the movement speed of the soil block unit on the prototype bucket be v = v_2_. If the displacement of the soil block relative to the bucket is *s*, then the kinetic energy of the soil block with respect to the bucket per unit time *t* on the surface of the bionic corrugated bucket is:

(16) $$\begin{array}{cc} M_{s}V_{0} & =m_{s}V_{1}+F_{bi}t\\ & =m_{s}V_{1}+[\tau_{x}l(B+S)+\mu_{f}N]t \end{array}$$

where *M_s_* is the mass of the bucket and *m_s_* is the mass of the soil cell.

For the prototype bucket:

(17) $$\begin{array}{cc} M_{s}V_{0} & =m_{s}v_{2}+F_{pr}t\\ & =m_{s}v_{2}+[\tau_{x}L(B+S)+\mu_{f}N]t \end{array}$$

From Equations (13) and (14):

(18) $$M_{s}{(v}_{1}-v_{2})=(L-l)\tau_{x}(B+S)$$

Clearly, the contact length of the bionic structure with the soil on the bionic corrugated surface *l* is less than the contact length of the prototype bucket surface L, i.e., L − l > 0. From this, it can be derived that v_1_ − v_2_ > 0 and thus v_1_ > v_2_. This indicates that the movement speed of the soil block on the bionic corrugated surface is greater than that on the prototype bucket surface. Consequently, on the bionic corrugated surface, soil particles can pass through the contact portion more quickly. The corrugated grooves produce an air film, reducing the contact area, minimizing adhesion, and achieving drag reduction.

### 4.6. Analysis of Bionic Circular Arc Drag Reduction Mechanism

**Figure 21:**
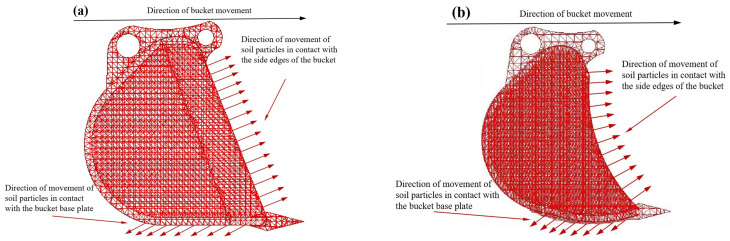
Schematic diagram of the movement direction of the soil particles in contact with the side edge, bottom plate, and soil. (**a**) Prototype bucket particle trajectory, (**b**) Bionic bucket particle trajectory.

One key factor for the drag reduction observed in the bionic arc structure is its ability to alter the motion direction of soil particles upon contact. This alteration disperses pressure, thereby reducing drag. Figure 21 schematically illustrates the motion direction of soil particles when the base plate and side edges of both the prototype bucket and the coupled bionic bucket-3 (C-B-3) are in contact with the soil. As the bucket moves to the right, the cutting effect of the prototype bucket’s side edges causes soil particles to move in a direction roughly perpendicular to the side edges. In contrast, the bionic side edges of the coupled bionic bucket-3 (C-B-3) feature a circular arc structure, causing the soil particles in contact to move in varied directions, dispersing the pressure. Additionally, as the bucket moves rightward, soil particles in contact with the bottom plate tend to move backward. The bionic rounded bottom plate further induces soil particles to move in various directions. When the bucket moves in an arc, this multi-directional movement of soil particles becomes more pronounced.

## 5. Conclusions

This study leverages the efficient digging behaviors of earthworms and pangolins to design biomimetic buckets. We extracted the anterior, middle, and posterior parts of the contraction-state curve of the earthworm head, along with the inner and outer contour curves of the pangolin claw toe. Using these curves, seven types of biomimetic buckets were designed. We then employed discrete element software to construct a soil–bucket interaction model and conducted simulation analyses at three different digging speeds. The following conclusions were drawn from the study:

(1) Determination of the properties of the soil revealed that 46.71 per cent of the soil particles were larger than 2 mm in size and that the shear modulus of the soil particles used was 1.05 × 10^6^.
(2) Compared to the prototype bucket, the bionic bucket exhibits lower excavation resistance, demonstrating excellent drag reduction performance. The drag reduction rate of the bionic bucket increases as the excavation speed decreases. The single-factor bionic bucket, based on the organism’s structure, shows significantly better drag reduction than the prototype bucket. Furthermore, the double-coupled bionic bucket outperforms the single-factor bionic bucket in terms of drag reduction. Among the designs, the coupled bionic bucket-3 (C-B-3) achieves the best drag reduction, with a maximum reduction rate of 14.469%.
(3) Different buckets induced varying levels of perturbation in soil particles, with the coupled bionic bucket-3 (C-B-3) demonstrating the highest efficiency in soil perturbation. Excavation speed significantly impacts the potential energy of soil particles; at an excavation speed of 2.5 rad/s, the potential energy increases by approximately 30% compared to at 2.0 rad/s. When the excavation speed reaches 3.0 rad/s, the potential energy further increases by about 25% compared to at 2.5 rad/s.
(4) An analysis of the drag reduction mechanisms of the bionic corrugated and arc structures reveals that the bionic corrugated surface facilitates the formation of an air film at the contact interface between the soil and the bucket. The presence of corrugated bumps allows soil particles to quickly pass through the contact area, significantly reducing the contact area and thereby achieving viscosity and drag reduction. Additionally, the bionic arc structure of the base plate and side edges causes soil particles in contact to move in different directions, dispersing pressure and further enhancing the reduction of viscosity and drag.

## Figures and Tables

**Table 1:** Soil particle size distribution.

Particle Size (mm)	>2	2~1	1~0.5	0.5~0.25	0.25~0.075	0.02~0.075	<0.02
Weight	467.08	68.23	32.15	164.67	23.50	99.01	145.36
Percentage %	46.71	6.823	3.215	16.467	2.35	9.901	14.536

**Table 2:** Different curves and geometric features.

Geometric Figure	Main Feature	Equation Sequence Number	*F* (*x*) Value
the front part of the contractile-state curve of the earthworm head	Different function curves of the inner and outer contours	f (x_1_)	0.4sinx
the middle and back part of the contractile-state curve of the earthworm head		f (x_2_)	0.25sin2x
the outer contour curve of the pangolin claw	Different corrugated curves at front and middle and rear	f (x_3_)	−0.139x^2^ + 0.91x + 0.65
the inner contour curve of the pangolin claw		f (x_4_)	−0.128x^2^ + 1.08x + 0.95

**Table 3:** Test results of accumulation angle measurement.

Measured Item	1	2	3	4	5	Mean Value
Stacking Angle (°)	27.26	29.85	29.82	30.31	31.81	29.81

**Table 4:** Initial range of microscopic parameters.

Argument	Primary Range
Soil–soil collision recovery coefficient-A	0.15–0.75
Soil–soil rolling recovery coefficient-B	0.32–1.04
Soil–soil static friction coefficient-C	0–0.2
Soil–steel collision recovery coefficient-D	0.2–0.5
Soil–steel rolling recovery coefficient-E	0.5–1.2
Soil–steel static friction coefficient-F	0–0.2
JKR surface energy (J/m^3^)-G	1–5

**Table 5:** Plackett–Burman test design scheme and results.

Serial Number	A	B	C	D	E	F	G	Stacking Angle (°)
1	0.75	1.04	0.2	0.2	0.5	0	5	43
2	0.15	1.04	0	0.5	1.2	0	5	17.01
3	0.15	1.04	0.2	0.5	0.5	0	1	26.47
4	0.75	1.04	0	0.5	1.2	0.2	1	12.41
5	0.75	0.32	0.2	0.5	1.2	0	1	13.67
6	0.75	0.32	0.2	0.5	0.5	0.2	5	36.76
7	0.75	0.32	0	0.2	1.2	0	5	13.77
8	0.75	1.04	0	0.2	0.5	0.2	1	11.92
9	0.15	0.32	0	0.2	0.5	0	1	0
10	0.15	0.32	0.2	0.2	1.2	0.2	1	36.60
11	0.15	0.32	0	0.5	0.5	0.2	5	18.16
12	0.15	1.04	0.2	0.2	1.2	0.2	5	55.78

**Table 6:** Box–Behnken test factors and level table.

Level	Factor			
	B Soil–Soil Rolling Recovery Coefficient	D Soil–Steel Collision Recovery Coefficient	E Soil–Steel Rolling Recovery Coefficient	F Soil–Steel Static Friction Coefficient
−1	0.32	0.2	0.5	0
0	0.68	0.35	0.85	0.1
1	1.04	0.5	1.2	0.2

**Table 7:** Design and results of Box–Behnken experiment.

Serial Number	B	D	E	F	Stacking Angle (°)
1	0.68	0.5	1.2	0.1	36.58
2	0.68	0.2	0.85	0.2	39.69
3	0.68	0.2	1.2	0.1	36.13
4	0.68	0.35	0.85	0.1	34.02
5	0.68	0.35	0.5	0.2	33.43
6	0.68	0.35	0.85	0.1	34.68
7	1.04	0.35	1.2	0.1	36.13
8	0.32	0.5	0.85	0.1	25.174
9	0.32	0.35	1.2	0.1	27.79
10	0.68	0.5	0.5	0.1	30.37
11	0.68	0.35	0.5	0	23.27
12	0.32	0.35	0.85	0.2	26.75
13	0.32	0.2	0.85	0.1	32.21
14	0.68	0.35	0.85	0.1	32.42
15	0.68	0.35	0.85	0.1	29.68
16	1.04	0.35	0.85	0.2	32.21
17	1.04	0.2	0.85	0.1	32.17
18	0.32	0.35	0.85	0	19.44
19	0.68	0.5	0.85	0	27.07
20	0.68	0.35	0.85	0.1	32.25
21	1.04	0.35	0.85	0	29.55
22	0.68	0.2	0.5	0.1	25.22
23	0.68	0.35	1.2	0.2	28.90
24	0.68	0.2	0.85	0	22.29
25	1.04	0.35	0.5	0.1	27.52
26	1.04	0.5	0.85	0.1	33.43
27	0.68	0.35	1.2	0	25.55
28	0.68	0.5	0.85	0.2	31.63
29	0.32	0.35	0.5	0.1	23.90

**Table 8:** Soil parameter.

Arguments	Parameter Value
Soil trough size (length × width × height)/(mm × mm × mm)	800 × 300 × 300
Particle size dimensions	3
Soil particle density ρ_1_/(kg/m^3^)	1300
Poisson ratio of soil particles γ_1_	0.38
Shear modulus of soil particles G_1_/pa	1.05 × 10^6^
Steel density ρ_2_/(kg/m^3^)	7800
Poisson’s ratio of steel γ_2_	0.3
Shear modulus of steel particles G_2_/pa	7.27 × 10^10^
Soil–soil collision recovery coefficient *e*_1_	0.15
Soil–soil rolling recovery coefficient *e*_2_	0.68
Soil–soil static friction coefficient *e*_3_	0.2
Soil–steel collision recovery coefficient *f*_1_	0.35
Soil–steel rolling recovery coefficient *f*_2_	0.85
Soil–steel static friction coefficient *f*_3_	0.1
Acceleration of gravity g/(m·s^2^)	9.80
JKR surface energy	1

**Table 9:** Average resistance value and drag reduction rate of different buckets.

	Factors	Spatulas	Resistance Value (N)			Damping Rate (100%)		
Spatulas			2 rad/s	2.5 rad/s	3 rad/s	2 rad/s	2.5 rad/s	3 rad/s
		P-B	79.243	85.061	89.243			
Single-Factor Bionic Bucket		B-S	73.755	81.171	87.901	+6.926	+4.573	+2.411
		B-B	72.515	83.506	85.878	+8.49	+1.828	+3.771
		B-C-1	74.345	82.058	88.731	+6.181	+3.53	+0.574
		B-C-2	78.634	82.851	88.649	+0.768	+2.594	+0.666
Coupled Bionic Bucket		C-B-1	68.229	77.334	81.126	+13.899	+9.084	+9.095
		C-B-2	71.072	76.371	85.677	+10.311	+10.216	+3.995
		C-B-3	67.777	74.094	80.494	+14.469	+12.893	+9.804

**Table 10:** Average torque value of different buckets.

	Factors	Spatulas	Number	Torque Value (N·m)		
Spatulas				2 rad/s	2.5 rad/s	3 rad/s
		Prototype bucket	P-B	1.396	1.496	1.575
Single-Factor Bionic Bucket		Bionic side-blade bucket	B-S	1.059	1.13	1.175
		Bionic base-plate bucket	B-B	1.32	1.362	1.482
		Bionic corrugated bucket-1	B-C-1	1.133	1.332	1.439
		Bionic corrugated bucket-2	B-C-2	1.377	1.499	1.523
Coupled Bionic Bucket		Coupled bionic bucket-1	C-B-1	1.153	1.156	1.497
		Coupled bionic bucket-2	C-B-2	1.13	1.208	1.312
		Coupled bionic bucket-3	C-B-3	0.958	1.189	1.364

## Data Availability

The data presented in this study are available on request.

## Abbreviations

B-S bionic side-blade bucket; B-B bionic bottom bucket; B-C-1 bionic corrugated bucket-1; B-C-1 bionic corrugated bucket-2; C-B-1 coupled bionic bucket-1; C-B-2 coupled bionic bucket-2; C-B-3 coupled bionic bucket-3; P-B prototype bucket.
